# Patient satisfaction with information on oral anticancer agent use

**DOI:** 10.1002/cam4.1239

**Published:** 2017-11-23

**Authors:** Christel C. L. M. Boons, Lonneke Timmers, Natasja M. van Schoor, Eleonora L. Swart, N. Harry Hendrikse, Jeroen J. W. M. Janssen, Jacqueline G. Hugtenburg

**Affiliations:** ^1^ Cancer Center Amsterdam Department of Clinical Pharmacology and Pharmacy VU University Medical Center Amsterdam The Netherlands; ^2^ Amsterdam Public Health research institute VU University Medical Center Amsterdam The Netherlands; ^3^ Department of Epidemiology and Biostatistics VU University Medical Center Amsterdam The Netherlands; ^4^ Cancer Center Amsterdam Department of Radiology and Nuclear Medicine VU University Medical Center Amsterdam The Netherlands; ^5^ Cancer Center Amsterdam Department of Hematology VU University Medical Center Amsterdam The Netherlands

**Keywords:** Cancer, illness perception, medication adherence, medication information, oral anticancer agents, patient beliefs, patient education, patient satisfaction, quality of life

## Abstract

Adequate information on oral anticancer agent (OACA) use is an essential element of optimal cancer care. The present study aimed to get insight into the experiences of patients with information on OACA treatment and their characteristics regarding information dissatisfaction. Patients of four Dutch university hospitals using OACA participated in this observational study and completed the Satisfaction with Information about Medicines Scale (SIMS), EORTC Quality of Life Questionnaire‐C30, Brief Illness Perception Questionnaire, and Beliefs about Medicines Questionnaire‐Specific. Logistic regression analyses were used to determine factors associated with dissatisfaction with information. Patients (*n* = 208) using capecitabine (35%), lenalidomide (15%), imatinib (14%), temozolomide (12%), sunitinib (11%), thalidomide (5%), dasatinib (4%), erlotinib (2%), and nilotinib (2%) participated. Information on the following SIMS‐items was inadequate: how OACA elicit their effect, how long it takes before treatment works, how to conclude that treatment is effective, the risk of side effects and its management, interference with sex life, drowsiness, interference with other medication and alcohol and what to do in case of a missed dose. Younger age, hematological malignancy, dyspnoea, positive perception of consequences of the cancer, low perception of treatment control, and indifferent attitude towards OACA were associated with dissatisfaction with information. In conclusion, a considerable number of patients would have appreciated receiving more information on specific issues relating to the consequences of OACA treatment such as the effects and side effects of OACA and the interference of treatment with various aspects of their daily life. Oncologists, hematologists, lung‐oncologists and pharmacists may reconsider the provision of information on OACA treatment.

## Introduction

In hematology and oncology, the number of available oral drugs is rapidly increasing 1. Since 2000 over 40 new oral anticancer agents (OACA) have become available while numerous OACA are in clinical development. Whereas some OACA have replaced intravenous therapies or offer alternative treatment options, a substantial number of OACA has been specifically developed for the treatment of malignancies for which hitherto no effective therapy was available. As the result, the survival of patients with several types of cancer is increasing considerably 2.

Since OACA are generally taken at home, patients themselves are largely responsible for using their anticancer medication as prescribed. Similar to patients with oral medication for the treatment of chronic diseases, they may however have difficulties with adhering to OACA treatment. Using a variety of measurement methods, adherence rates ranging from 40% to 100% have been reported for different cancer therapies 3. Complexity of the dosing regimen can negatively influence the correct use of OACA 3, 4, 5. Notably in the case that comedication is used for the treatment of a chronic disease, this may cause confusion or lead to organizational problems 6, 7. The occurrence of side effects is also a factor prominently affecting adherence and even may cause patients to deliberately not take their mediation as prescribed in order to minimize discomfort 8.

Adequate information on OACA is therefore an essential element of optimal cancer care. Although varying between diseases and patients, patients with cancer have a clear need for information in all phases of their disease 9, 10, 11, 12. They should not only have sufficient knowledge about the way to correctly use their OACA, but also about the various effects that these drugs bring about 4. Indeed, adequate information has been positively related to medication adherence 13, 14 and quality of life 12, 15, 16. Addressing concerns about OACA treatment and helping patients to understand the importance of their treatment is essential in achieving optimal adherence 17. However, data on patient satisfaction with information regarding OACA use is scarce 14, 18 and little is known about factors related to (dis‐)satisfaction. The present study aimed to get insight into the experiences of cancer patients with information on their treatment with OACA and their characteristics regarding dissatisfaction with the information provided. These data will be useful for optimizing patient information on OACA provided by healthcare providers (HCP).

## Material and Methods

### Study design

An observational, cross‐sectional, multicentre study 19 was conducted in four Dutch academic hospitals: VU University Medical Centre Amsterdam (VUmc), Leiden University Medical Centre (LUMC), Radboud University Medical Centre Nijmegen (Radboudumc) and University Medical Centre Groningen (UMCG). Data were collected between October 2010 and March 2012 by means of a composite questionnaire (see Measures). The study was approved by the Medical Ethics Review Board of VUmc as well as the boards of the participating hospitals.

### Patients

A 3‐month period of the pharmacy databases of the outpatient pharmacies of the participating hospitals was screened for patients. Patients who had filled at least one prescription for an OACA (i.e. capecitabine, dasatinib, erlotinib, everolimus, gefitinib, imatinib, lapatinib, lenalidomide, nilotinib, sorafenib, sunitinib, temozolomide, or thalidomide) were extracted. Exclusion criteria were: too ill to participate, age younger than 18 years, inability to fill out a questionnaire and insufficient Dutch language skills. Both patients on treatment and patients off treatment were eligible for participation. Written informed consent was obtained from all patients who participated.

### Measures

#### Demographic and treatment characteristics

Patients completed a self‐administered composite questionnaire. The questionnaire started with various questions on demographic data including age, gender, education, living status, and work status. Education was assessed as the highest level completed, ranging from elementary education to university. The variable was dichotomized into higher education (higher general secondary education or above) and lower education. Living status was assessed as living alone or not living alone, and work status as having paid work or not. Data on malignancy (solid tumour vs. hematological disease), dosing regimen (cyclic vs. continuous), and duration of treatment were retrieved from the medical files and/or pharmacy dispensing records.

#### Satisfaction with information on OACA treatment

The main outcome of the study was patients’ experience with information on OACA treatment using the Dutch version of the validated Satisfaction with Information about Medicines Scale (SIMS) 13. SIMS evaluates the extent to which patients feel that they have been given adequate information on prescribed medicines. The questionnaire consists of 17 items, each referring to a particular aspect of medicine use. Patients were asked to rate the information they were provided using the following response scale: “too much”, “about right”, “too little”, “none received”, “none needed”. Satisfaction with information (ratings “about right” or “none needed”) was given a score of one. Dissatisfaction (ratings “too much”, “too little”, or “none received”) was scored as zero. Ratings for each item were examined to identify specific information that patients felt they had not been provided sufficiently. An *overall satisfaction rating* was obtained by summing the scores of all 17 items (score ranges from 0–17). A higher score indicates a higher degree of satisfaction with information. The scale scores showed good internal reliability with a Cronbach's alpha coefficient of 0.87. Summing items 1–9 identifies satisfaction with information on *action and usage* (score ranges from 0 to 9) (Cronbach's alpha 0.79); items 10–17 identify satisfaction with information on *potential problems* (score ranges from 0 to 8) (Cronbach's alpha 0.80) 13.

#### Quality of life

Quality of life was only assessed in patients on treatment. The European Organization for Research and Treatment of Cancer Quality of Life Questionnaire Core 30 (EORTC QLQ‐C30) was used to evaluate quality of life 20. The 30‐items questionnaire incorporates a *global health status*, five functional scales (*physical, role, emotional, cognitive,* and *social*), three symptom scales (*fatigue, nausea/vomiting,* and *pain*), five single items assessing additional symptoms commonly reported by cancer patients (*dyspnoea, insomnia, appetite loss, constipation,* and *diarrhea*) and one question about the *financial impact* of the disease. Each item was scored on a 4‐point scale (1 = not at all to 4 = very much). The scales and single item raw scores were linear transformed according to the original scoring manual into a standardized score ranging from 0 to 100 20. A higher score indicates better health and functioning, or denotes more pain and symptoms.

#### Illness perception

Illness perception was assessed using the Brief Illness Perception Questionnaire (Brief IPQ) 21. The Brief IPQ evaluates the cognitive and emotional representations of an illness. Eight items were scored on a continuous linear scale from zero to 10 (i.e. *consequences, time line, personal control, treatment control, concern, identity, coherence,* and *emotional response)*. A higher score indicates a stronger perception of the item. Items *personal control, treatment control,* and *identity* were reversed prior to score calculation.

#### Beliefs about OACA treatment

Patient beliefs about OACA treatment were assessed using the validated Beliefs about Medicines Questionnaire (BMQ) 22. In the present study, only the part BMQ‐Specific focusing on patient beliefs on the medication prescribed, was used. It includes the BMQ‐Specific *necessity* which measures beliefs of the necessity of taking a medicine for controlling the illness (i.e. that both present and future health depends on the medicine and that it prevents a worsening of the disease) and the BMQ‐Specific *concerns* which measures concerns about the use of a medicine (i.e. worries about side effects, long‐term effects, and becoming addicted). Each item of the two 5‐item subscales is scored using a 5‐point Likert scale (1 = strongly disagree to 5 = strongly agree) resulting in a score for the subscales ranging from five to 25 per scale. A higher score indicates stronger beliefs in the concepts represented by the scale. An indication of the relative importance of the necessity beliefs and concerns is obtained by calculating the necessity‐concerns differential: the difference between *necessity* and *concerns* scores, ranging from −20 to 20. If the difference is positive, the patient perceives that the necessity of the medicine prescribed outweighs the concerns about its use. Patients were categorized into four attitudinal groups: accepting (high necessity, low concerns), ambivalent (high necessity, high concerns), indifferent (low necessity, low concerns) and skeptical (low necessity, high concerns) with the scale midpoint of 15 or above used as a cut‐off to define high beliefs 23.

### Statistics

Characteristics of the study population have been described as frequencies (percentages) for categorical variables, means and standard deviation (SD) for normally distributed continuous variables and medians and quartiles for skewed continuous variables. Logistic regression analyses were used to determine influencing factors for dissatisfaction versus satisfaction with information as assessed by SIMS. Only patients on treatment (*n* = 130) were included in the logistic regression analyses because quality of life was not assessed in patients off treatment. The median scores of the three SIMS scales were used to define dissatisfaction (<14 of 17 items, <8 of 9 items, and <6 of 8 items for, respectively, *overall satisfaction rating*, subscale *action and usage* and subscale *potential problems)*. Because the distribution of the three SIMS scales was left‐skewed, the scales were dichotomized into dissatisfaction (coded as 1) versus satisfaction (0). Univariable logistic regression analyses were performed relating the different factors to the three SIMS scales. All factors with *P* ≤ 0.157 (Akaike Information Criterion) in the univariable analyses were included in a multivariable logistic model 24. A backward elimination procedure was used where at each step the variable with the highest *P*‐value was removed from the models until only variables with *P* < 0.10 remained. The relationship with age and most of the items on quality of life and illness perception was nonlinear. Age was therefore categorized into 18–55, 56–69, and ≥70 years. Items on quality of life and illness perception were dichotomized into the most adverse quartile versus the other three quartiles. Data from the dispensing records of the pharmacy were used to compare the responders with the nonresponders. Age was compared using a *t*‐test and gender and OACA were compared using Chi‐Square test. For all analyses, a two‐tailed significance level of 0.05 was used. *P*‐values below this level were considered statistically significant. Statistical analysis was performed with SPSS 22 for Windows (IBM Corp, Armonk, NY, USA).

## Results

### Study sample

**Figure 1:**
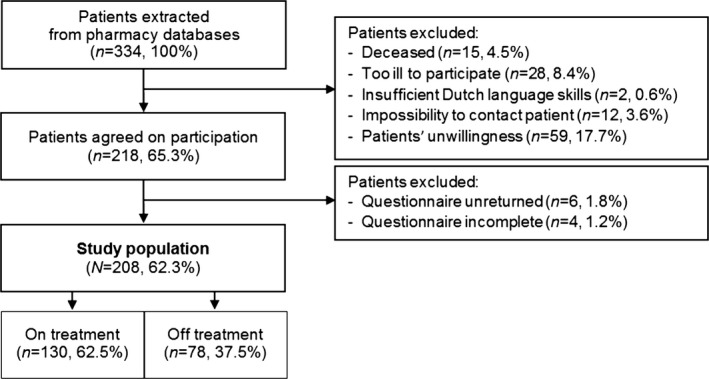
Flowchart of patient recruitment.

Figure 1 shows the patient selection, response and reasons for nonparticipation. The age and gender distributions of the study population (*n* = 208) and nonresponders (*n* = 126) were compared and revealed no significant differences. There were also no differences between the study population and nonresponders with regard to the OACA used (data not shown). The mean age of the participants was 58.5 years (SD 12.5) and 55% was male. Most patients (*n* = 130; 62.5%) were on treatment at the time of the study, 78 patients (37.5%) had discontinued treatment. Patient characteristics are shown in Table 1.

**Table 1:** Patient demographics and treatment characteristics (*N* = 208)

	On treatment (*n* = 130)	Off treatment (*n* = 78)
Patient demographics		
Age, *n* (%)		
18–55 years	54 (41.5%)	19 (24.4%)
56–69 years	52 (40.0%)	45 (57.7%)
≥70 years	24 (18.5%)	14 (17.9%)
Female gender, *n* (%)	61 (46.9%)	32 (41.0%)
Higher level of education, *n* (%)	59 (45.7%)	32 (41.0%)
Living alone, *n* (%)	24 (18.5%)	15 (19.2%)
Paid work, *n* (%)	36 (27.7%)	18 (23.1%)
Treatment characteristics		
Malignancy, *n* (%)		
Solid tumor	75 (57.7%)	63 (80.8%)
Hematological	55 (42.3%)	15 (19.2%)
Oral anticancer agent, *n* (%)		
Capecitabine	30 (23.1%)	42 (53.8%)
Dasatinib	7 (5.4%)	1 (1.3%)
Erlotinib	4 (3.1%)	1 (1.3%)
Imatinib	28 (21.5%)	2 (2.6%)
Lenalidomide	20 (15.4%)	11 (14.1%)
Nilotinib	4 (3.1%)	0 (0.0%)
Sunitinib	13 (10.0%)	9 (11.5%)
Temozolomide	15 (11.5%)	10 (12.8%)
Thalidomide	9 (6.9%)	2 (2.6%)
Dosing regimen, *n* (%)		
Cyclic	57 (43.8%)	11 (14.1%)
Continuous	73 (56.2%)	67 (85.9%)
Duration of treatment (days), M ± SD (range)		
Capecitabine	201 ± 200 (28–777)	226 ± 202 (47–1086)
Dasatinib	456 ± 318 (80–931)	431 ± NA (NA)
Erlotinib	415 ± 448 (26–1018)	1019 ± NA (NA)
Imatinib	899 ± 925 (60–3221)	1415 ± 1860 (100–2730)
Lenalidomide	306 ± 253 (70–993)	263 ± 200 (100–806)
Nilotinib	161 ± 87 (74–277)	NA
Sunitinib	574 ± 505 (69–1594)	400 ± 343 (101–1228)
Temozolomide	209 ± 256 (35–1081)	331 ± 246 (118–989)
Thalidomide	185 ± 148 (60–506)	297 ± 154 (188–406)

M, mean; SD, standard deviation.

### Satisfaction with information on OACA treatment

Table 2 shows the quality of the information on OACA treatment per SIMS item. Twenty‐three percent of patients were completely satisfied with all items. Most patients who were satisfied indicated that the information provided was about right. Few patients indicated that information was not needed (≤8% for each of the individual 17 SIMS‐items) or that they were given too much information (≤2%). In almost all cases of patients who reported to be dissatisfied, this resulted from having received too little or no information. In particular, information was deemed insufficient on how OACA elicit their effect (26%), how long it takes before treatment works (38%) and how to conclude that treatment is effective (45%), the risk of side effects (29%) and its management (24%), interference of OACA treatment with sex life (44%), possibility of drowsiness (37%), interference with other medication (30%) and the use of alcohol (29%). Information on what to do in case of a missed dose was considered insufficient in 29% of patients.

**Table 2:** Patient satisfaction with information on OACA treatment (Satisfaction with Information about Medicines Scale [SIMS]) (*N* = 208)

	Satisfied, %			Dissatisfied, %			
	About right	None needed	Total	Too much	Too little	None received	Total
What your medicine is called	88.9	3.4	92.3	0.0	4.3	3.4	7.7
What your medicine is for	90.9	2.4	93.3	0.0	5.8	1.0	6.8
What it does	84.6	1.0	85.6	0.0	11.1	3.4	14.5
How it works	71.6	1.9	73.5	1.0	17.3	8.2	26.5
How long it will take to act	57.0	3.9	60.9	1.0	16.4	21.7	39.1
How you can tell if it is working	48.5	5.8	54.3	0.5	19.4	25.7	45.6
How long you will need to be on your medicine	75.5	3.8	79.3	0.5	6.7	13.5	20.7
How to use your medicine	94.7	1.4	96.1	0.0	2.9	1.0	3.9
How to get a further supply	95.2	2.4	97.6	0.0	0.5	1.9	2.4
Whether the medicine has any unwanted effects (side effects)	80.7	1.0	81.7	1.9	12.6	3.9	18.4
What are the risks of you getting side effects	68.9	1.0	69.9	1.5	15.5	13.1	30.1
What you should do if you experience unwanted side effects	73.7	1.5	75.2	1.0	13.2	10.7	24.9
Whether you can drink alcohol whilst taking this medicine	63.8	7.2	71.0	0.0	10.1	18.8	28.9
Whether the medicine interferes with other medicines	64.1	5.3	69.4	0.5	12.6	17.5	30.6
Whether the medicine will make you feel drowsy	57.8	4.4	62.2	0.5	13.1	24.3	37.9
Whether the medicine will affect your sex life	46.6	8.3	54.9	1.0	6.9	37.3	45.2
What you should do if you forget to take a dose	66.7	4.3	71.0	0.5	11.6	16.9	29.0

OACA, oral anticancer agent.

Table S1 displays the medians and quartiles for the nine scales and six items of the EORTC QLQ‐C30 and the eight dimensions of the Brief IPQ as well as the means and SD for the three subscales of the BMQ‐Specific.

### Factors of patient dissatisfaction with information on OACA treatment

Tables 3 (univariable analyses) and 4 (multivariable analyses) explore the relationships between patient and treatment characteristics and information dissatisfaction. According to the multivariable analyses patients aged younger than 56 were more likely to be dissatisfied with the information provided on potential problems of OACA treatment as compared to 56–69‐year‐olds (OR 2.94, 95% CI 1.18–7.69). Patients using OACA for the treatment of a hematological malignancy were also more often dissatisfied with this information as compared to patients with solid tumors (OR 3.65, 95% CI 1.58–8.44). The experience of dyspnea was associated with dissatisfaction about information given on potential problems of OACA treatment (OR 4.79, 95% CI 2.02–11.33) as well as with the overall satisfaction rating (OR 2.36, 95% CI 1.10–5.07). With respect to illness perception, a low perception of treatment control was associated with dissatisfaction about information on potential problems of OACA treatment (OR 4.00, 95% CI 1.24–12.85) and a positive perception of the consequences of cancer with dissatisfaction about information on the SIMS action and usage items (OR 3.57 95% CI 1.20–10.00). With respect to beliefs about medication, patients with an indifferent attitude towards OACA (low necessity, low concerns) were more often dissatisfied with information on action and usage as compared to patients with an accepting attitude (high necessity, low concerns) (OR 1.69, 95% CI 1.02–2.81).

**Table 3:** Factors associated with patient dissatisfaction with information on OACA treatment (univariable analyses)

	Overall dissatisfaction with information			Dissatisfaction with information on action and usage			Dissatisfaction with information on potential problems		
	*n* a	OR (95% CI)	*P*‐value	*n* a	OR (95% CI)	*P*‐value	*n* a	OR (95% CI)	*P*‐value
Patient characteristics									
Age (years)									
18–55	54	1.08 (0.50–2.32)	0.837	54	1.18 (0.55–2.55)	0.673	54	2.00 (0.92–4.34)	**0.080**
56–69	52	*ref*		52	*ref*		52	*ref*	
≥70	24	0.75 (0.28–2.04)	0.573	24	0.89 (0.33–2.40)	0.811	24	1.75 (0.65–4.70)	0.270
Female gender	129	1.48 (0.74–2.96)	0.273	130	1.24 (0.61–2.49)	0.554	129	1.40 (0.70–2.80)	0.344
Higher level of education	128	1.03 (0.52–2.08)	0.925	129	0.71 (0.35–1.43)	0.335	128	1.12 (0.56–2.24)	0.754
Living alone	129	1.01 (0.41–2.45)	0.992	130	1.52 (0.63–3.71)	0.354	129	0.59 (0.24–1.46)	0.254
Paid work	129	0.80 (0.37–1.73)	0.564	130	1.01 (0.46–2.20)	0.985	129	0.70 (0.32–1.52)	0.367
Hematological cancer	129	1.74 (0.86–3.53)	**0.125**	130	1.16 (0.57–2.35)	0.678	129	2.49 (1.22–5.11)	**0.013**
Cyclic dosing regime	129	0.65 (0.32–1.31)	0.228	130	0.96 (0.48–1.94)	0.908	129	0.68 (0.34–1.36)	0.273
>1 year on treatment	129	0.79 (0.38–1.66)	0.532	130	0.66 (0.31–1.40)	0.280	129	0.91 (0.44–1.90)	0.803
Hospital									
1	37	*ref*		37	*ref*		37	*ref*	
2	22	1.64 (0.57–4.78)	0.362	22	2.22 (0.76–6.50)	**0.148**	22	0.66 (0.23–1.91)	0.438
3	51	1.35 (0.57–3.20)	0.496	51	1.06 (0.44–2.56)	0.892	51	0.84 (0.36–1.97)	0.691
4	19	2.26 (0.73–6.97)	**0.157**	19	2.05 (0.67–6.32)	0.211	19	1.05 (0.35–3.19)	0.928
Quality of life (EORTC QLQ‐C30)^a^									
Global health status	128	1.34 (0.56–3.22)	0.510	129	0.74 (0.30–1.82)	0.509	128	1.79 (0.74–4.35)	0.201
Functional scales									
Physical functioning	127	1.15 (0.50–2.63)	0.748	128	1.58 (0.69–3.60)	0.277	127	1.45 (0.63–3.33)	0.382
Role functioning	128	1.71 (0.71–4.12)	0.234	129	1.92 (0.80–4.56)	**0.142**	128	1.52 (0.63–3.66)	0.354
Emotional functioning	129	1.11 (0.53–2.34)	0.788	130	1.15 (0.55–2.43)	0.711	129	2.34 (1.09–5.04)	**0.030**
Cognitive functioning	129	1.55 (0.60–4.05)	0.368	130	0.55 (0.20–1.55)	0.260	129	2.97 (1.06–8.29)	**0.038**
Social functioning	128	1.96 (0.77–4.98)	0.158	129	1.66 (0.67–4.11)	0.272	128	2.80 (1.05–7.42)	**0.039**
Symptom scales									
Fatigue	128	1.34 (0.58–3.07)	0.490	129	1.17 (0.51–2.69)	0.713	128	2.05 (0.88–4.78)	**0.098**
Nausea and vomiting	129	1.06 (0.51–2.19)	0.877	130	0.91 (0.44–1.89)	0.796	129	1.59 (0.77–3.29)	0.214
Pain	129	1.09 (0.50–2.35)	0.833	130	0.94 (0.44–2.05)	0.884	129	0.95 (0.44–2.06)	0.905
Dyspnoea	129	2.59 (1.25–5.35)	**0.010**	130	1.76 (0.86–3.61)	**0.123**	129	3.35 (1.59–7.03)	**0.001**
Insomnia	129	1.70 (0.68–4.21)	0.255	130	1.36 (0.55–3.37)	0.501	129	1.88 (0.75–4.72)	0.179
Appetite loss	129	0.71 (0.34–1.52)	0.381	130	0.64 (0.29–1.37)	0.247	129	1.50 (0.71–3.17)	0.292
Constipation	129	1.37 (0.64–2.95)	0.418	130	1.40 (0.65–3.00)	0.387	129	1.40 (0.65–3.01)	0.389
Diarrhea	128	1.57 (0.28–1.19)	**0.132**	129	1.71 (0.54–5.42)	0.360	128	1.81 (0.56–5.86)	0.324
Financial difficulties	128	2.21 (0.98–4.96)	**0.055**	129	1.44 (0.65–3.18)	0.372	128	1.94 (0.87–4.35)	**0.107**
Illness perception (Brief IPQ)^a^									
Consequences	127	0.59 (0.25–1.41)	0.234	128	0.39 (0.15–1.00)	**0.051**	127	0.77 (0.33–1.78)	0.535
Time line	125	–	–	126	–	–	125	–	–
Personal control	126	1.10 (0.46–2.65)	0.825	127	1.37 (0.57–3.31)	0.479	126	0.65 (0.27–1.59)	0.349
Treatment control	127	2.07 (0.75–5.75)	0.162	128	2.54 (0.92–7.08)	**0.074**	127	2.45 (0.86–7.00)	**0.095**
Concern	127	1.25 (0.54–2.90)	0.603	128	0.74 (0.31–1.76)	0.490	127	0.92 (0.40–2.14)	0.848
dentity	129	1.63 (0.71–3.75)	0.249	130	1.42 (0.62–3.27)	0.405	129	2.08 (0.89–4.86)	**0.090**
Coherence	128	1.22 (0.51–2.88)	0.655	129	0.84 (0.35–2.02)	0.694	128	0.89 (0.38–2.11)	0.794
Emotional response	129	0.92 (0.42–2.01)	0.825	130	0.66 (0.29–1.48)	0.310	129	1.11 (0.51–2.43)	0.792
Beliefs about OACA (BMQ–specific)									
Attitudinal groups									
Accepting	72	*ref*		72	*ref*		72	*ref*	
Ambivalent	39	1.12 (0.51–2.45)	0.771	40	1.16 (0.53–2.55)	0.709	39	1.18 (0.54–2.57)	0.683
Indifferent	12	1.18 (0.35–4.02)	0.789	12	3.14 (0.87–11.42)	**0.082**	12	1.12 (0.33–3.80)	0.858
Sceptical	2	1.18 (0.07–19.64)	0.907	2	1.57 (0.09–26.15)	0.753	2	1.12 (0.07–18.57)	0.938

OACA, oral anticancer agent; EORTC QLQ‐C30, European Organization for Research and Treatment of Cancer Quality of Life Questionnaire Core 30; Brief IPQ, Brief Illness Perception Questionnaire; BMQ‐Specific, Beliefs about Medicines Questionnaire Specific; OR, odds ratio; 95% CI, 95% confidence interval. Variables with *P* ≤ 0.157 are shown in bold and were included in a multivariable logistic model (Table 4).

Variables were dichotomized into most adverse quartile versus the other three quartiles (reference category).

a Only patients on treatment (*n* = 130) were included in the regression analyses because quality of life was not assessed in patients off treatment.

**Table 4:** Factors associated with patient dissatisfaction with information on OACA treatment (multivariable analyses)

	Overall satisfaction with information			Satisfaction with information on action and usage			Satisfaction with information on potential problems		
	*n* a	OR (95% CI)	*P*‐value	*n* a	OR (95% CI)	*P*‐value	*n* a	OR (95% CI)	*P*‐value
Patient characteristics									
Age (years)									
18–55							51	2.98 (1.18–7.51)	**0.021**
56–69							51	*ref*	
≥70							22	1.88 (0.62–5.63)	0.262
Hematological cancer							124	3.65 (1.58–8.44)	**0.003**
Quality of life (EORTC QLQ‐C30)^a^									
Functional scales									
Role functioning				123	2.37 (0.88–6.36)	0.088			
Symptom scales									
Dyspnea	128	2.36 (1.10–5.07)	**0.027**				124	4.79 (2.02–11.33)	**<0.001**
Diarrhea	128	0.48 (0.22–1.04)	0.064						
Financial difficulties	128	2.36 (0.97–5.72)	0.058						
Illness perception (Brief IPQ)^a^									
Consequences				123	0.28 (0.10–0.83)	**0.022**			
Treatment control				123	2.61 (0.87–7.77)	0.086	124	4.00 (1.24–12.85)	**0.020**
Beliefs about OACA (BMQ‐specific)									
Attitudinal groups									
Accepting				72	*ref*				
Ambivalent				38	1.09 (0.47–2.53)	0.840			
Indifferent				11	6.03 (1.29–28.28)	**0.023**			
Skeptical				2	0.70 (0.04–12.41)	0.811			

Univariable variables with *P* ≤ 0.157 were included in the multivariable logistic model. Significant relations are shown in bold (*P* < 0.05). Abbreviations: OACA, oral anticancer agent; EORTC QLQ‐C30, European Organization for Research and Treatment of Cancer Quality of Life Questionnaire Core 30; Brief IPQ, Brief Illness Perception Questionnaire; BMQ‐Specific, Beliefs about Medicines Questionnaire Specific; OR, odds ratio; 95% CI, 95% confidence interval.

Variables were dichotomized into most adverse quartile versus the other three quartiles (reference category).

a Only patients on treatment (*n* = 130) were included in the regression analyses because quality of life was not assessed in patients off treatment.

## Discussion

In the present study, most patients were satisfied with the information provided as far as it concerned instructions on the use of OACA and their supply. However, a considerable number would have appreciated receiving more information on specific issues such as the effects and side effects of the OACA they used and the interference of treatment with various aspects of their daily life. Dissatisfaction with information was related to younger age, the presence of a hematological malignancy, dyspnea, a positive perception of the consequences of cancer, a low perception of treatment control, and an indifferent attitude towards OACA.

Information insufficiency particularly concerned OACA actions and effectiveness, (management of) side effects, interference of OACA treatment with sex life, possible occurrence of drowsiness, interference with other medication, the use of alcohol and what to do in case of a missed dose. In the present study, a third of the patients used capecitabine. Data on patient satisfaction with information on OACA use are limited to the results of a study performed in UK 18 and a study performed in India 14, and mainly concern the use of capecitabine. Similar to the results of the present study, in these studies considerable dissatisfaction originated from a lack of information about the self‐monitoring of treatment effectiveness, i.e. how OACA elicit their effect, how long it takes before treatment works and how to conclude that treatment is effective 14, 18. The insufficiency regarding information on the self‐monitoring of effectiveness has been explained by the unpredictability of the onset of the action and somatic experience of capecitabine treatment 18. Subsequently, satisfaction with information might be increased by informing patients in more general terms and explaining that the effect of the treatment in individual patients is highly unpredictable.

Inadequate information on side effects has been described as one of the most prominent unmet information needs of cancer patients 9, 11, 12. In the present study, inadequate information on side effects included the risk of their occurrence and their management. However, in the UK study 18, including patients using capecitabine, a lack of information about side effects was not reported. Patients may have received more information about side effects, because the majority of the patients experienced side effects 18. In the present study patients were particularly concerned about drowsiness. Since drowsiness is not a common side effect of OACA, caregivers intentionally and as such correctly, may have omitted to provide information on this specific side effect. Nevertheless, given the extent of concern, it is clear that adequate information on (social) issues which impact daily routines of patients like side effects, the use of alcohol or the interference of OACA treatment with a patient's sex life, is an important unmet need that should be addressed by caregivers.

Although problems about sexuality are widespread among cancer patients, information on this subject is often not provided 9, 11, 25, 26, 27. In the present study, 44% of the patients considered themselves inadequately informed on this issue. Similar to drowsiness, side effects related to sexuality as such are not commonly attributed to OACA use and information on this subject may therefore have been omitted. However, sexual functioning and intimacy may also be affected indirectly as the result of OACA treatment side effects like fatigue, hair loss and weight gain or effects directly caused by the malignancy or its treatment such as organ loss or scarring 28. Providing information on this topic may also be difficult for both caregivers and patients 25. However, with almost half of the patients being dissatisfied with information concerning sexuality, it is clear that, either for contraceptive purposes 29 or for quality of life, more attention is needed for this subject.

Thirty percent of patients missed information on the interference of OACA with other medicines. Drug‐drug interactions in cancer treatment might be associated with serious or even fatal adverse events, or may weaken the therapeutic effect 30. Since cancer patients often use several medicines as part of their anticancer treatment or for the management of side effects or comorbidities, adequate information on drug‐drug interactions with OACA is essential.

Similar to the studies on the use of capecitabine 14, 18, a considerable number of patients indicated not to have received information on what to do in the case of a missed dose. One‐fifth of the patients in the present study indicated to have sometimes forgotten to take their OACA 19. This stresses the need to explain in a more explicit manner how to deal with missed doses.

Younger patients (18–55‐year‐olds vs. 56–69‐year‐olds) were more likely to be dissatisfied with the information on potential problems of OACA treatment. Younger patients may have higher information demands than older patients 9. However, no association was found with the patients aged 70 years and older. Patients with hematological malignancies were also more likely to be dissatisfied with the information than patients with solid tumors. This is in contradiction with the results of a review 11, which concluded that the majority of the patients with hematological malignancies were satisfied with the provided information. The level of satisfaction might also relate to the difference in information provision between hospitals. However, in the present study variations in dissatisfaction with information could not be attributed to hospital differences. With respect to the influence of side effects, only dyspnea was found to be a predictor for information dissatisfaction. Side effects and their consequences are an important aspect of quality of life and have been found to relate to information satisfaction 12, 15, 16. It is unclear why only for dyspnea a significant relationship with information dissatisfaction was found and not for the experience of any other side effects of the EORTC QLQ‐C30 questionnaire.

The results of the present study indicate that patient beliefs are important when considering patient satisfaction with information. Patients with a more positive perception of the *consequences* of cancer were more likely to be dissatisfied with information on the action and usage of their treatment, while patients with a low perception of *treatment control* were more likely to be dissatisfied with information on potential problems of OACA. Furthermore, an indifferent attitude towards OACA, including rather low concerns and low necessity beliefs (vs. an accepting attitude) was associated with information dissatisfaction. In other studies, it was also observed that the beliefs of cancer patients influence information satisfaction 18, 31, 32. However, study results are not consistent. Satisfaction with information has been associated with better illness perception on all IPQ subscales 31, 32, except for the items *personal control* 31 and *identity* 32. With respect to patient beliefs about medication, patients in the UK study 18 with stronger concerns about their medicines were less satisfied with information. Two‐thirds of the patients in the present study were treated with other OACA than capecitabine, but did not differ from the patients using capecitabine with respect to their beliefs about OACA (data not shown).

There are some strengths and limitations to this study. First, while previous studies have been performed in a single hospital 14, 18, the present study was conducted in four centers and is one of the largest realized about this topic. This increases the representativeness of the present study. Second, detailed information on the extent of (dis‐)satisfaction with information on OACA treatment was identified by using the SIMS questionnaire. Strength of the SIMS is that it gauges patients’ own beliefs about the information provided, rather than measuring the absolute quantity or quality of the information 13. Another strength of this study is the wide range of factors explored to determine which patients are more likely to be dissatisfied with information. In spite of the strength of the patient‐reported method to measure information on OACA treatment, it is unclear whether the information was actually provided or which type of HCP was involved. Nevertheless, the information should have been perceived as sufficient, regardless actual provided information and its source.

## Conclusion

Although most patients were provided sufficient general information on their treatment with OACA, a considerable number indicated to have appreciated more information on specific issues such as the effects and side effects of their OACA and the interference of treatment with various aspects of their daily life. Variations in dissatisfaction with information on OACA use could be attributed to age, malignancy, the experience of dyspnea, and patient beliefs.

## Practice Implications

A substantial number of patients perceived a lack of information on OACA treatment. This deficiency needs to be addressed by extending information or providing information in a more effective manner. Particularly information on the following subjects should be improved: the self‐monitoring of treatment effectiveness, side effects and their management, interference of treatment with sex life, interference of treatment with other medicines and alcohol use, and how to act in case of missed doses. Oncologists, hematologists, lung‐oncologists and pharmacists may reconsider the provision of information on OACA treatment.

## Conflict of Interest

The authors report no conflicts of interest.

## Supporting information


**Table S1.** Quality of life, illness perception and patients’ beliefs about OACA.
