# Supplementary material for: Patient satisfaction with information on oral anticancer agent use
Source: Cancer Med. 2017 Nov 23;7(1):219–28. doi: 10.1002/cam4.1239 (PMC5773956; doi:10.1002/cam4.1239)
Supplement: Supplementary file 1 — Table S1. Quality of life, illness perception and patients’ beliefs about OACA. [file CAM4-7-219-s001.docx]

| **Supplementary table S1: Quality of life, illness perception and patients’ beliefs about OACA** | | | | |
| --- | --- | --- | --- | --- |
|  | range | Q1 | Median | Q3 |
| **Quality of life (EORTC QLQ-C30)(n=130)** |  |  |  |  |
| Global health status | 0-100 | 58 | 67 | 83 |
| Functional scales |  |  |  |  |
| Physical functioning | 0-100 | 67 | 80 | 93 |
| Role functioning | 0-100 | 50 | 67 | 100 |
| Emotional functioning | 8-100 | 67 | 83 | 100 |
| Cognitive functioning | 0-100 | 67 | 83 | 100 |
| Social functioning | 0-100 | 67 | 83 | 100 |
| Symptom scales |  |  |  |  |
| Fatigue | 0-100 | 22 | 33 | 56 |
| Nausea and vomiting | 0-83 | 0 | 0 | 17 |
| Pain | 0-100 | 0 | 0 | 33 |
| Dyspnoea | 0-100 | 0 | 0 | 33 |
| Insomnia | 0-100 | 0 | 33 | 33 |
| Appetite loss | 0-100 | 0 | 0 | 33 |
| Constipation | 0-100 | 0 | 0 | 33 |
| Diarrhoea | 0-100 | 0 | 0 | 33 |
| Financial difficulties | 0-100 | 0 | 0 | 33 |
| **Illness perception (Brief IPQ)(n=208)** |  |  |  |  |
| Consequences | 0-10 | 6 | 8 | 9 |
| Time line | 0-10 | 7 | 10 | 10 |
| Personal control | 0-10 | 1 | 4 | 7 |
| Treatment control | 0-10 | 7 | 8 | 10 |
| Identity | 0-10 | 3 | 6 | 8 |
| Concern | 0-10 | 5 | 7 | 9 |
| Coherence | 0-10 | 4 | 7 | 9 |
| Emotional response | 0-10 | 2 | 5 | 7 |
|  | range | Mean ± SD | | |
| **Beliefs about OACA (BMQ-Specific)(n=208)** |  |  | | |
| Subscales |  |  | | |
| Necessity | 6-25 | 19.2 ± 4.1 | | |
| Concerns | 5-25 | 13.5 ± 3.9 | | |
| Necessity-Concerns differential | -10-20 | 5.7 ± 5.4 | | |
| Attitudinal groups, % |  |  | | |
| Ambivalent | 49.8% | | | |
| Accepting | 37.4% | | | |
| Indifferent | 9.9% | | | |
| Sceptical | 3.0% | | | |
| Abbreviations: OACA, oral anticancer agent; EORTC QLQ-C30, European Organization for Research and Treatment of Cancer Quality of Life Questionnaire Core 30; Brief IPQ, Brief Illness Perception Questionnaire; BMQ-Specific, Beliefs about Medicines Questionnaire Specific; Q1, 25th percentile; Q3, 75th percentile; SD, standard deviation. | | | | |
